# Generalized Cohen’s d for Multiple Means and Polytomous Settings

**DOI:** 10.1177/01466216261416025

**Published:** 2026-01-20

**Authors:** Jari Metsämuuronen

**Affiliations:** 1 Turku Research Institute for Learning Analytics (TRILA), Faculty of Mathematics and Natural Sciences, 8058 University of Turku, Turku, Finland

**Keywords:** effect size, Cohen’s *d*, Cohen’s *f*, eta squared, generalized Cohen’s *d*

## Abstract

Cohen’s *d* is the most commonly used estimator to quantify the magnitude of the difference between the means of two subpopulations. When comparing multiple populations simultaneously, Cohen’s *f* can be used for the same purpose. Using their relationship in the dichotomous setting, several general formulas for *d* are derived that generalize *d* to the polytomous setting. The traditional simplified estimator *d* = 2*f* is studied as a shortcut estimator. It is strongly recommended to use the general formulas instead of the simplified ones when assessing the magnitude of the effect size, especially when the discrepancy of the extreme proportions of cases in the subpopulations exceeds 0.40.

## Introduction

Effect size (ES) is a concept related to the quantitative measurement of the magnitude of a phenomenon of interest (usually the difference in the group means or the strength of the relationship between two variables) in a population, or a sample-based estimate of that magnitude (e.g., Kelley & Preacher, 2012), or, as Cumming and Calin-Jageman (2017, p. 111) put it simply: ES “is the amount of anything that’s of research interest.” The rationale for using effect sizes is that the traditional statistical inference related to statistical significance or *p*-value is strictly dependent on the sample size. However, the magnitude of the difference between the means or of the association may be trivially small even though it may be “true” or the “most likely” in the population. Therefore, the leading journals in empirical education and psychology began in the late 1980s and early 1990s to encourage the reporting of some measure of effect size in addition to statistical significance (see the history in Huberty, 2002; Peng & Chen, 2014). Based on statistics from Kirk (1996) and Schäfer (2018), Schäfer and Schwarz (2019) note that the number of articles in prominent APA journals reporting inferential statistics *and* effect sizes increased from 48% to nearly 100% in 20 years.

Peng and Chen (2014) assess that the three most commonly reported measures for ES are the unadjusted *R*^2^, Cohen’s *d*, and *η*^2^ while in the Schäfer and Schwarz (2019) dataset, the most common are Pearson’s r, Cohen’s *d*, and partial eta squared, $\eta_{p}^{2}$ . Of these, eta squared is closely related to Cohen’s *f*, an estimator closely related to *d*. Other estimators are typologized by Huberty (2002) and Peng and Chen (2014), among others. Among the “families” of estimators of ESs, the family based on relationship includes such estimators as product-moment correlation (PMC, *r*), coefficient of determination (*r*^2^, *R*^2^), eta squared (*η*^2^) as well as derivatives such as Cohen’s *f* and *f*^2^. The family of group differences includes such estimators as Cohen’s *d*, standardized mean difference (*θ*), Glass’ delta, and Hedge’s *g*. However, the classifications are not strict. Cohen’s *f* and *d* can be located in either group, since both can be expressed in terms of correlations or by test statistics related to between-group differences.

Traditionally, Cohen’s *d* is restricted to dichotomous cases with two subpopulations (Cohen, 1969, 1988). A comparable estimator for the polytomous cases with more than two subpopulations is Cohen’s *f*. In the dichotomous cases, these two estimators are closely related, but not in the form of *f* that we usually see in textbooks. The truly comparable forms are discussed below. It may be worth recalling that the traditional verbal descriptions “small,” “medium,” and “large” (Cohen, 1969, 1988) extended by “very small,” “very large,” and “huge” (Sawilowsky, 2009) for *f* as being *half* of that by *d* are based on simplified form of the relationship between *f* and *d*, that is, *d* = 2*f* (Cohen, 1988, p. 276). This is true only in the special case where the number of cases in both groups is equal. The same is true for the *r* effect size thresholds. The actual effect sizes and thresholds can be remarkably different when the comparable formulas are used. These are discussed later on in the article.

Cohen’s *d* is a well-known and widely used estimator of ES, and the thresholds associated with the qualitative thresholds of the magnitude of the difference between the means and the magnitude of the point-biserial correlation (*R*_*PB*_) are well known and (presumably) generally accepted. From this perspective, it is somewhat unsatisfactory that *d* is limited to settings with two means (*f*) or two categories (*R*_*PB*_). In particular, the acceptance associated with the traditional thresholds does not apply to the point-polyserial correlation or to the correlation between two continuous variables (see. e.g., Funder & Ozer, 2016; Gignac & Szoborai, 2016). It seems that Cohen’s transformation formula (Cohen, 1988, p. 82; Cohen, 1992), which is based on equal sample sizes in the dichotomous cases and a simplified relationship between point-biserial (PB) and biserial (BS) correlation, that is, *R*_*PB*_ = 1.253 × *R*_*BS*_, gives us too high value for the “medium” and “large” effect sizes. Instead of Cohen’s standards 0.1, 0.3, and 0.5 for “small,” “medium,” and “large” effect sizes, respectively, the thresholds should be closer to 0.1, 0.2, and 0.3 (cl., Funder & Ozer, 2016; Gignac & Szoborai, 2016 from the empirical point of view, and Metsämuuronen, 2024c from the theoretical-empirical point of view).

In what follows, the link between *d* and *f* is used to derive a generalized form for *d* that provides a comparable estimate of ES in both in the dichotomous and polytomous settings. The advantage of such a general form is that the verbal attributes related to ESs and their numerical values correspond between Cohen *f* and Cohen *d* regardless of the discrepancy between the number of cases in the subpopulations or the number of categories.

The study commences with an examination of the comparable forms of *d* and *f*. In this section, the applied users of *f* and *r* effect sizes are provided with refined thresholds for binary and dichotomous settings that also take into account the discrepancy between the group sizes. This broadens the view of the traditional simplified thresholds for “small,” “medium,” and “large” effect sizes based on the assumption of equal numbers of cases in the subpopulations. Several general forms of *d* are then derived. Finally, two options for shortcut estimators are discussed and their properties are studied using a simulated data set based on a real-world setting.

## Relation of Cohen’s *d* and Cohen’s *f* in the Dichotomous Settings

### Comparable Forms of *d* and *f*

Consider a nominal or ordinal variable *g* with observations *x*_*i*_, *R* subpopulations, each with *n*_*i*_ number of cases in subpopulation *i*, and a metric (ordinal, interval or continuous) variable *X* with observations *y*_*i*_ across *C* categories. In the context of *d* and *f*, we are usually interested in quantifying the differences between the group means (*μ*_*i*_) with respect to *X*. In the context of *d* and *R*, we consider the same settings from the point of view of the number of categories in *g*; that is, for *d* and *R*_*PB*_, two categories are of interest, while for *d* and *R*_*PP*_, several ordinal categories are of interest. Later, the general symbol for both of these is *R*_*gX*_. In the case of continuous or semi-continuous variables, also discussed in the article, the number of categories in the variables is the same, that is, we are interested in the relationship of *d* and *ρ*_*XY*_.

Cohen’s classic book introducing the concepts associated with the conventional standards for interpreting effect sizes (conceptualized in 1962, originally published in 1969, revised in 1977 and completed in 1988) gives many forms for *d* depending on different settings with different assumptions. The book is organized so that the simplified forms, which assume equal group sizes and equal variances in the subpopulations are given first and the general, more complicated forms are given the last, or are only hinted at. For example, the general form of *d* is only hinted at on page 44 with the comment “*Under these conditions* ( $\sigma_{A}\neq \sigma_{B}$ *and* $n_{A}\neq n_{B}$ , *simultaneously*, *the values […] may be greatly in error*,” and the general form of *f* is half given on page 359 after 80 pages of discussion of the simplified formulas.

Metsämuuronen (2024d) discusses in detail the incomparable and comparable forms of the formulas for transforming *r* and *f* to the scale of *d* and vice versa. Some formulas that are relevant from the point of view of this article are highlighted here. Since the coefficient eta is equal to point-biserial correlation in the dichotomous setting (see, e.g., Metsämuuronen, 2022a, 2023a) and since *f* is defined by eta squared as $f=\eta_{g|X}/\sqrt{1-\eta_{g|X}^{2}}$ (Cohen, 1988), *d* can be expressed in the binary and dichotomous settings as follows:

(1) $$d_{1}=\frac{\eta_{g|X}}{\sqrt{1-\eta_{g|X}^{2}}}\times \left(\frac{n_{1}+n_{2}}{\sqrt{n_{1}\times n_{2}}}\right)=\sqrt{\frac{\eta_{g|X}^{2}}{1-\eta_{g|X}^{2}}}\times \frac{1}{\sqrt{p_{1}p_{2}}}=\frac{f}{\sqrt{p_{1}p_{2}}}=\frac{f}{\sqrt{p_{i}\left(1-p_{i}\right)}},$$

(derived from Cohen, 1988, p. 24), where *p*_*i*_ refers to one of the proportions of the two subpopulations, and eta squared is traditionally calculated as follows:

(2) $$\eta_{g|X}^{2}=\frac{\overset{R}{\underset{i=1}{\sum }}n_{i}{\left(\mu_{i}-\mu_{X}\right)}^{2}}{\overset{R}{\underset{i=1}{\sum }}\overset{C}{\underset{j=1}{\sum }}{\left(x_{ij}-\mu_{X}\right)}^{2}}=\frac{\overset{R}{\underset{i=1}{\sum }}p_{i}{\left(\mu_{i}-\mu_{X}\right)}^{2}}{\sigma_{X}^{2}},$$

where $\mu_{X}$ and $\sigma_{X}^{2}$ are the grand mean and variance of *X*, respectively. It may be worth noting that equation (1) does not, in fact, produce estimates that are fully consistent with *d* when the coefficient eta is computed in the conventional way. Namely, by using the traditional way to estimate the coefficient eta, equation (1) does not capture the negative values that are relevant to *d*, indicating which groups had lower means. The reason is that the conventional way of calculating the coefficient eta is based on first calculating eta squared as in equation (2), and then taking the square root. This procedure truncates all the negative values of eta to positive values.

For the dichotomous settings, we have a form that would correctly produce the negative values (see, e.g., Metsämuuronen, 2022a). For the comparable estimates in both dichotomous and polytomous ordinal settings, the form suggested by Metsämuuronen (2022a, 2023a), which also allows negative values, is as follows:

(3) $$d_{3}=sign\left(R_{gX}\right)\times \frac{\eta_{g|X}}{\sqrt{\left(1-\eta_{g|X}^{2}\right)}}\times \frac{1}{\sqrt{p_{1}p_{2}}}=sign\left(R_{gX}\right)\times \frac{f}{\sqrt{p_{1}p_{2}}}=sign\left(R_{gX}\right)\times \frac{f}{\sqrt{p_{i}\left(1-p_{i}\right)}}$$

(Metsämuuronen, 2022a), where $sign\left(R_{gX}\right)$ refers to the sign of PMC between an ordinal *g* and a metric *X*. In particular, this correction does not make sense for truly nominal categories, since PMC does not make sense between nominal and ordinal variables. However, it always makes sense in the dichotomous, ordinal, and interval settings.

Notably, Cohen (1988) does not discuss these forms, but gives a well-known simplified form *d* = 2*f* which is the result when *p*_1_ = *p*_2_ = 0.5, that is, when we assume or observe equal group sizes.

### Refined Thresholds of *d* and *f* in the Dichotomous Settings

Knowing that the traditional thresholds for “small,” “medium,” and “large” effect sizes are given as 0.2, 0.5, and 0.8 for *d* and as 0.1, 0.25, and 0.4 for *f*, respectively, (Cohen, 1988, pp. 284–288), we note that the latter thresholds are exact only when the group sizes are equal (*p*_*i*_ = 0.5), leading to a simplified form of *f* = 0.5*d*. In many practical settings, the numbers of cases in the subpopulations differ from each other and, assuming that the thresholds for *d* are the benchmarks, the true thresholds for *f* are much *lower* than those given by Cohen (1988).

Table 1 collects the “true” or refined thresholds by selected proportions of cases in the subpopulations for the applied user. We note, for example, that *f* = 0.26 is traditionally considered to reflect a “medium” effect size although it should be considered to reflect a “very large” effect size if either of the groups contains only 5% of the cases ( $d=0.26/\sqrt{0.05\left(1-0.05\right)}=1.19$ ). Note that these “refined” thresholds are not new ones but based on Cohen’s original formulas. They are used as benchmarks for the polytomous settings.

**Table 1.** Refined thresholds for Cohen f for selected proportions of group sizes based on equation (1)

		Cohen’s d					
		0.1	0.2	0.5	0.8	1.2	2
	p_1_ = *n*_1_/(*n*_1_ + *n*_2_)	“Very small”^b^	“Small”^a^	“Medium”^a^	“High”^a^	“Very high”^b^	“Huge”^b^
Cohen’s f	0.5	0.05	0.10	0.25	0.40	0.60	1.00
	0.4	0.05	0.10	0.24	0.39	0.59	0.98
	0.3	0.05	0.09	0.23	0.37	0.55	0.92
	0.2	0.04	0.08	0.20	0.32	0.48	0.80
	0.1	0.03	0.06	0.15	0.24	0.36	0.60
	0.05	0.02	0.04	0.11	0.17	0.26	0.44

^a^Cohen (1988).

^b^Sawilowsky (2009).

## Cohen’s *d* With Multiple Means and in the Polytomous Settings

### Generalized Formulae for Cohen’s *d* for the Multiple Means

One challenge with *d* that is relevant from the perspective of this article is that it is limited to two-population settings. Metsämuuronen (2024c) discusses the case of polytomous settings from the point of view of *r* effect size. Here, the case of multiple means is discussed. When comparing multiple means, *f* is a parallel estimator to *d*. An estimate of the effect size by *f* is easily calculated when eta squared is available (see equations (1) and (3)).

Using the same logic as in deriving a general formula for transforming product-moment correlation estimates to the scale of *d* (Metsämuuronen, 2024c), we can reasonably assume that equation (1) is in fact a *reduced form* of a more general form of *d*, although we do not know what the general formula would be in the polytomous setting. A plausible guess is that the form might take the following form:

(4) $$d_{4}=\frac{f}{A\times \sqrt{\frac{1}{R\left(R-1\right)}\sum_{i=1}^{R}p_{i}p}}$$

where the element $\overset{R}{\underset{i=1}{\sum }}p_{i}p_{j}$ generalizes the element *p*_1_*p*_2_ of equation (1) to multiple means and polytomous settings, and we compute the average of all possible combinations of *p*_*i*_*p*_*j*_. The element *R* (*R* – 1) refers to the number of the elements *p*_*i*_*p*_*j*_ to be averaged.^1^ The element *A* is an element needed to adjust the transformation by the number of groups in some form.

While we know from equation (1) that the reduced form in the case of *R* = 2 has the form $d_{1}=f/\sqrt{p_{1}p_{2}}$ , the element *A* in equation (4) must have the form

(5) $$A=\frac{R}{2}$$

To obtain the reduced form of the form in the dichotomous settings with *R* = 2. Then, because of (1), (4), and (5), the general form of *d* for the polytomous settings is as follows:

(6) $$d_{6}=\sqrt{\frac{\eta_{g|X}^{2}}{1-\eta_{g|X}^{2}}}/\sqrt{\frac{R}{4\left(R-1\right)}\overset{R}{\underset{i=1}{\sum }}p_{i}p_{j}}=f/\sqrt{\frac{R}{4\left(R-1\right)}\overset{R}{\underset{i=1}{\sum }}p_{i}p_{j}}.$$

When *R* = 2, equation (6) takes the form $d_{6}=f/\sqrt{\frac{2}{4\cdot \left(2-1\right)}\cdot \left(p_{1}p_{2}+p_{2}p_{1}\right)}$ = $f/\sqrt{p_{1}p_{2}}=d_{1}$ .

Combining equations (6) and (3), a form that is relevant in dichotomous and ordinal settings that produces both negative and positive estimates is as follows:

(7) $$d_{7}=sign\left(R_{gX}\right)\times f/\sqrt{\frac{R}{4\left(R-1\right)}\overset{R}{\underset{i=1}{\sum }}p_{i}p_{j}}=sign\left(R_{gX}\right)\times \sqrt{\frac{\eta_{g|X}^{2}}{1-\eta_{g|X}^{2}}}/\sqrt{\frac{R}{4\left(R-1\right)}\overset{R}{\underset{i=1}{\sum }}p_{i}p_{j}}.$$

From equation (1) we get a hint that the element $\overset{R}{\underset{i=1}{\sum }}p_{i}p_{j}$ could also be $\overset{R}{\underset{i=1}{\sum }}p_{i}\left(1-p_{i}\right)$ . This leads to another form of the general estimator. The latter element can be manipulated as follows (and spaces are left where a particular term is missing):

(8) $$\begin{array}{l} \overset{}{\underset{}{\sum_{i=1}^{R}}}p_{i}\left(1-p_{i}\right)=p_{1}\left(p_{2}+p_{3}+...+p_{R}\right)\\ +p_{2}\left(p_{1}+p_{3}+...+p_{R}\right)\\ +p_{3}\left(p_{1}+p_{2}+p_{4}+...+p_{R-1}+p_{R}\right)\\ ...\\ +p_{R-1}\left(p_{1}+p_{2}+p_{3}+p_{4}+...+p_{R}\right)\\ +p_{R}\left(p_{1}+p_{2}+p_{3}+p_{4}+...+p_{R-1}\right) \end{array}$$

By opening the elements, we get the following form, where some of the identical terms are highlighted:

(9) $$\begin{array}{l} \overset{R}{\underset{i=1}{\sum }}p_{i}\left(1-p_{i}\right)=\underline{p_{1}p_{2}}+\underset{‗}{p_{1}p_{3}}+p_{1}p_{4}...+p_{1}p_{R-1}+p_{1}p_{R}\\ +\underline{p_{2}p_{1}}+p_{2}p_{3}+...+p_{2}p_{R-1}+p_{2}p_{R}\\ +\underset{‗}{p_{3}p_{1}}+p_{3}p_{2}+p_{3}p_{4}+...+p_{3}p_{R-1}+p_{3}p_{R}\\ ...\\ +p_{R-1}p_{1}+p_{R-1}p_{2}+p_{R-1}p_{3}+...+p_{R-1}p_{R-2}+\underline{p_{R-1}p_{R}}\\ +p_{R}p_{1}+p_{R}p_{2}+p_{R}p_{3}+...+p_{R}p_{R-2}+\underline{p_{R}p_{R-1}}\\ =\overset{R}{\underset{i=1}{\sum }}p_{i}p_{j} \end{array}$$

Then, because of equations (8) and (9),

(10) $$\overset{R}{\underset{i=1}{\sum }}p_{i}\left(1-p_{i}\right)=\overset{R}{\underset{i=1}{\sum }}p_{i}p_{j}.$$

Consequently, from (1), (6), and (10), we obtain an alternative form for the general *d* as follows:

(11) $$d_{11}=\sqrt{\frac{\eta_{g|X}^{2}}{1-\eta_{g|X}^{2}}}/\sqrt{\frac{R}{4\left(R-1\right)}\overset{R}{\underset{i=1}{\sum }}p_{i}\left(1-p_{i}\right)}=f/\sqrt{\frac{R}{4\left(R-1\right)}\overset{R}{\underset{i=1}{\sum }}p_{i}\left(1-p_{i}\right)}.$$

Equation (11) always yields positive estimates. Combining equations (11) and (3), an alternative form for the ordinal settings that also produces negative values is as follows:

(12) $$\begin{array}{l} d_{12}=sign\left(R_{gX}\right)\times f/\sqrt{\frac{R}{4\left(R-1\right)}\overset{R}{\underset{i=1}{\sum }}p_{i}\left(1-p_{i}\right)}\\ =sign\left(R_{gX}\right)\times \sqrt{\frac{\eta_{g|X}^{2}}{1-\eta_{g|X}^{2}}}/\sqrt{\frac{R}{4\left(R-1\right)}\overset{R}{\underset{i=1}{\sum }}p_{i}\left(1-p_{i}\right)} \end{array}.$$

In the very case that the group sizes are identical, all the general formulas above get the form *d* = 2*f*. Namely, if *n*_*i*_ = *n*_*j*_, $p_{i}=1/R$ . By substituting *p*_*i*_, we get

(13) $$\overset{R}{\underset{i=1}{\sum }}p_{i}\left(1-p_{i}\right)=\overset{R}{\underset{i=1}{\sum }}\left(p_{i}-p_{i}^{2}\right)=\overset{R}{\underset{i=1}{\sum }}p_{i}-\overset{R}{\underset{i=1}{\sum }}p_{i}^{2}=1-Rp_{i}^{2}=1-\frac{1}{R}=\frac{R-1}{R}.$$

Then,

(14) $$\frac{R}{4\left(R-1\right)}\overset{R}{\underset{i=1}{\sum }}p_{i}\left(1-p_{i}\right)=\frac{R}{4\left(R-1\right)}\times \frac{\left(R-1\right)}{R}=\frac{1}{4}.$$

Consequently, with the same number of cases in the subpopulations, *d*_6_ = *d*_11_ and *d*_7_ = *d*_12_, and all equal with $f/\sqrt{\frac{R}{4\left(R-1\right)}\overset{R}{\underset{i=1}{\sum }}p_{i}\left(1-p_{i}\right)}=f/\sqrt{\frac{1}{4}}$ = 2*f* regardless of the number of subpopulations.

Obviously, all the estimators *d*_6_, *d*_7_, *d*_11_, and *d*_12_ give identical absolute values for the estimates. In the following, they are expressed as giving “exact” estimates. This needs to be considered in relation to the shortcut estimators which approximate the “exact” estimate. Of course, keep in mind that we are ultimately estimating the *population* effect size, and then the estimate based on a sample can never give absolutely “exact” estimates.

### An Alternative as Shortcut Estimators for the Generalized *d*

Manual calculation of the previous forms can be a bit tedious in applied settings when the number of groups is very large. Therefore, shortcuts can be valuable for practical use. In this section, the traditional simplified estimator discussed above, *d* = 2*f*, is studied as a shortcut to approximate the exact estimate.

#### Research Question

The question is how well do the estimates produced by the shortcut estimator agree with the exact estimates produced by the general formulas for Cohen’s *d* in the polytomous settings?

#### Dataset Used in the Simulation

A published dataset with 14,880 estimates of effect sizes based on nationally representative test-takers of a mathematics test (FINEEC, 2018) is used to model the fit between the estimates by the shortcut estimators and the exact but more complicated estimators of the generalized Cohen *d*. The dataset is available in CSV format at https://doi.org/10.13140/RG.2.2.20359.57762/1 and in IBM SPSS format at https://doi.org/10.13140/RG.2.2.33781.35042/1. The characteristics and peculiarities of the dataset are discussed in detail in Appendix 1 (see also Metsämuuronen, 2022a, 2022b, 2023a).

Although the data set is actually related to measurement modeling settings with items (*g*) and scores (*X*), we can think of the data as consisting of different (ordinal) conditions of proportions of subpopulations with respect to some interesting metric dependent variable *X*, such as attitudes or achievement. The ordinal nature of the grouping variables does not affect the results because the underlying estimator of correlation, the coefficient eta, does not use this information. The dataset contains few negative estimates of eta that could have been used in the analysis. However, all of the negative estimates appear to come from the binary settings, so they are not useful in the modeling.

The data set is somewhat specific, including variables with high item-score correlations and a mechanical relationship between each item and the score variable, as is always the case in measurement modeling settings. These do not affect the calculation of the Cohen’s *f* estimates and the examination of the simplified estimators, since the underlying coefficient eta does not use the information about the mechanical relationship of the variables. However, it does affect the fact that items with more categories are more highly correlated with the metric variable than items with fewer categories. This will be seen and commented on in some of the following plots. The main effect is that the estimates are relatively high. In particular, no traditional verification mechanism was applicable in the data set used, due to its particular characteristics. Therefore, it is suggested that different types of datasets be used to verify or dispute the results regarding the behavior of the shortcut methods.

#### Discrepancy Index Related to the Deviance in the Number of Cases in the Subpopulations

Above, in the binary or dichotomous settings, it was noted that the success of transforming the estimates of *f* to the scale of *d* depends on the element $\sqrt{p_{1}p_{2}}$ , which reflects the discrepancy between the proportions of the cases in the subpopulations. For the polytomous settings, another type of indicator is used for the same purpose. It is called here the discrepancy index *p*_*d*_. It is simply the absolute difference between the highest and lowest proportions of cases in the groups, that is, *p*_*d*_ = *p*_max_ − *p*_min_, where *min* and *max* refer to the groups with the highest and lowest number of cases, respectively. For example, suppose we have four groups to compare with the following proportions of cases in the subpopulations: *p*_A_ = 0.20, *p*_B_ = 0.24, *p*_C_ = 0.52, and *p*_D_ = 0.04. The maximum proportion is *p*_max_ = 0.52 and the minimum proportion is *p*_min_ = 0.04. Then, *p*_*d*_ = 0.52–0.04 = 0.48.

If the highest and lowest proportions are equal, *p*_*d*_ = 0. This is the case, where the simple transformation formula *d* = 2*f* gives accurate estimates. In the simulation data set, the discrepancy index for the polytomous items varies *p*_d_ = 0.11–0.84 with a mean of $\bar{p_{d}}$ = 0.41 (*SD* = 0.13).

#### Simplified Formula *d* = 2*f* as an Option for a Shortcut Estimator

The traditional simplified formula for transforming Cohen’s *f* estimates to the scale of Cohen’s *d* is as follows (Cohen, 1988, p. 276):

(15) $$d_{15}=2f$$

It is clear from Table 1 that the higher is the discrepancy index, the less the estimates from the simplified formula correspond to the true values. However, we do not know how noticeable this underestimation is in the polytomous settings associated with comparing multiple means.

**Figure 1.**
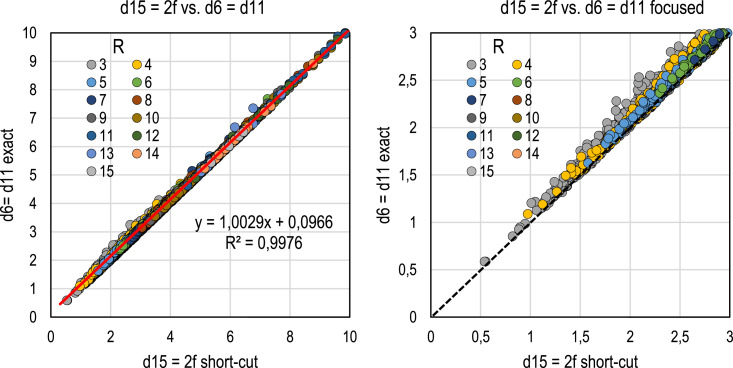
Relationship between *d*_15_ = 2*f* and the exact estimate in polytomous cases

**Figure 2.**
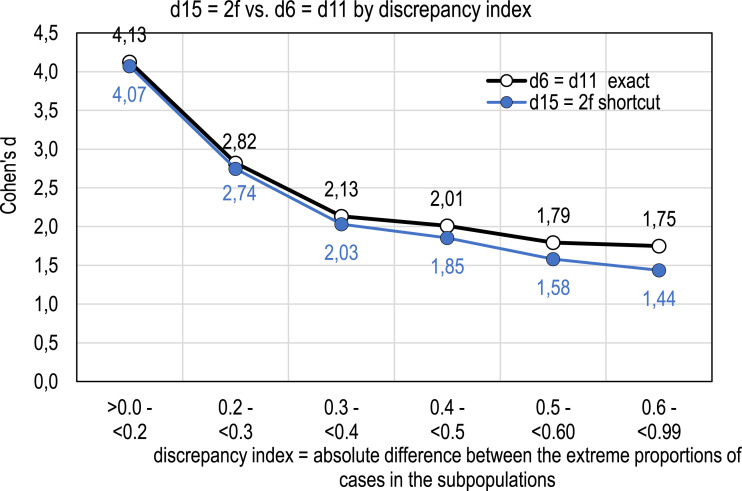
Relationship between the estimates from *d*_15_ = 2*f* and the exact estimates; means

Based on the 6,932 polytomous estimates of eta squared in the simulation dataset, the shortcut estimator *d*_15_ = 2*f* is highly correlated with the true values (*R*^2^ = 0.998), and the estimates are *always lower in magnitude* than the exact ones as expected (Figure 1). However, the estimates tend to be quite close to the exact values when the group sizes are close to each other (Figure 2).

Two points are worth noting from Figures 1 and 2. First, when the absolute difference between the highest and lowest proportion of group sizes is small or moderate (*p*_*d*_ < 0.30 on average), the estimates from *d*_15_ tend to underestimate the effect size by less than 0.08 units of *d*. When *p*_*d*_ exceeds 0.30, the discrepancy could be described as “huge” in Sawilowsky’s terms; the average difference exceeds 0.10 units of *d*. When one group dominates the group sizes in terms of magnitude (*p*_*d*_ > 0.60), the underestimation tends to be more than 0.30 units of *d*.

Second, the characteristic that the estimates tend to become larger in magnitude as the number of groups increases and the discrepancy decreases (see Figure 2) is caused by the specificities in the simulation data set. Because of the mechanical correlation between the item and score, the higher is the number of categories in *g* the higher is the correlation between *g* and *X*. This has a strong effect on the magnitude of the coefficients eta and eta squared, and thus on the magnitude of *f*. At the same time, the discrepancy between the proportions of group sizes gets smaller the wider the scale is; with a wide scale the cases tend to be more evenly distributed than with a narrow scale, especially with small sample sizes. This determines the size of the discrepancy index.

In summary, while representing a highly simplified formula, the traditional equation for transforming *f* to the scale of *d* (*d*_15_ = 2*f*) provides a surprisingly accurate approximation of the generalized *d*, even when the number of cases in the subpopulations differs. However, it has a tendency to systematically underestimate the true value. It also produces broadly comparable estimates in comparison with the more general estimators (*d*_6_ and *d*_11_) when the discrepancy in the proportions of the largest and smallest group sizes is small or moderate (*p*_*d*_ < 0.30). For a very large discrepancies between the largest and smallest numbers of cases in the groups (*p*_*d*_ > 0.40), an estimate with *d*_15_ may radically underestimate the true effect size. It is not known how well this shortcut would work in general data sets. Therefore, results with the simplified estimator *d = 2f* are preliminary, and systematic studies are needed to confirm its usefulness in general settings.

### Numerical Example of Computing Cohen’s *d* in a Polytomous Setting

Suppose we are interested in comparing the performance levels of students in three groups with respect to the intensity of support needed to learn. The statistics related to such a setting are summarized in Table 2. The statistics are published and based on a national assessment of learning outcomes in mathematics in Finland (Metsämuuronen, 2023d). From Table 2 it is known that 86% of the students received general support from the teacher while 10% received additional intensified support and 4% received special support also from a specialist teacher. The discrepancy index gets the value *p*_*d*_ = 0.8587 − 0.0408 = 0.8179, which indicates a radical discrepancy between the number of cases in the subpopulations. In this case, we expect a radical underestimation by the shortcut estimators.

**Table 2.** Statistics for estimating generalized Cohen’s *d* in a real-life settings

Subpopulation	Group	Mean	Std. Deviation	*N*	p_i_	pi (1 − pi)	pipj
0	General support	468.7	107.3593	10.493	0.8587	0.1213	*p*_0_*p*_1_: 0.0863
1	Intensified support	358.9	91.7799	1.228	0.1005	0.0904	*p*_0_*p*_2_: 0.0350
2	Special support	332.9	98.4396	498	0.0408	0.0391	*p*_1_*p*_2_: 0.0041
	Total	452.2	113.2576	12.219			

	Equation	Eta squared	R_gX_
		0.1317	−0.351
		Cohen d	Effect size in a verbal form
*d*_6_ = *d*_11_ exact	6, 11	1.2699	“Very large”
*d*_7_ = *d*_12_ exact	7, 12	−1.2699	“Very large”
*d*_15_ = 2*f* shortcut	15	0.7789	“Medium” or “large”

The smaller groups are for those students who have been identified as needing more support due to learning difficulties. Thus, we *expect* to see lower achievement levels in the special support groups (the actual significance level in the original data set is, of course, *p* < .001 due of the large sample size), but the magnitude of the effect is of particular interest to us. In general, we can ask in the traditional way, “how remarkable is the difference between the group means,” or in a more technical way: “how much does the grouping variable reduce the free information in the data set.” The latter might be a reasonable interpretation for the estimates of *f* and the generalized *d*. The wording is based on the information criteria (IC) familiar from the structural equation modeling (SEM). IC describes the amount of information or “order” that the model brings. In a fully independent (or worst possible) model there is a lot of free information or “disorder” compared with the saturated (or best possible) model. A large effect size reflects the fact that the model (i.e., the partitioning of the data into these specific categories) notably or remarkably reduces the amount of free information or disorder in the data set.

The interest is to compare the result with the general estimators (*d*_6_ = *d*_11_ and *d*_7_ = *d*_12_) with the “exact” estimates, with a shortcut estimator (*d*_15_ = 2*f*) with approximations. Since the relationship between performance and the ordinal grouping variable is negative (the less support needed the better the results), the estimators that produce negative estimates of *f* and *d* (*d*_7_, *d*_12_) are also reported in Table 2.

Eta squared is given (0.1317), and it implies that the underlying eta is $\sqrt{0.1319}=0.3629$ . Consequently, $f=\sqrt{0.1317/\left(1-0.1317\right)}=0.3896$ . The latter should actually be negative estimates, as indicated by the negative sign of the PMC (*R*_*gX*_ = −0.351). The negative sign makes sense: the more support is needed the lower the level of mathematics achievement.

The statistics needed to calculate *d*_6_ and *d*_7_ are obtained as follows: $p_{0}p_{1}=p_{1}p_{0}=0.8587\times 0.1005=0.0863$ , $p_{0}p_{2}=p_{2}p_{0}=0.8587\times 0.0408$ = 0.0350, and $p_{1}p_{2}=p_{2}p_{1}=0.1005\times 0.0408=0.0041$ . The statistics for the calculation of *d*_11_ and *d*_12_ are as follows: $p_{0}\left(1-p_{0}\right)=0.8587\times \left(1-0.8587\right)=0.1213$ , $p_{0}\left(1-p_{0}\right)=0.1005\times \left(1-0.1005\right)=0.0904$ , and $p_{2}\left(1-p_{2}\right)=0.0408\times \left(1-0.0408\right)=0.0391$ .

The estimates by *d*_6_ and *d*_11_ are computed as follows:

$$d_{6}=d_{11}=0.3895/\sqrt{\frac{3}{4\times 2}\times \left(2\times 0.0863+2\times 0.0350+2\times 0.0041\right)}=1.2699$$

or

$$d_{6}=d_{11}=0.3895/\sqrt{\frac{3}{4\times 2}\times \left(0.1213+0.0904+0.0391\right)}=1.2699.$$

Correspondingly, the estimates by *d*_7_ and d_12_ are computed as follows:

$$d_{7}=d_{12}=-0.3895/\sqrt{\frac{3}{4\times 2}\times \left(0.1213+0.0904+0.0391\right)}=-1.2699.$$

All indicate a “very large” effect size by Sawilowsky’s standards.

The traditional simplified formula for transforming *f* to the scale of *d* gives the following estimate: *d*_15_ = 2 × 0.3895 = 0.7789 which indicates a “large” effect size. In this case, the transformation leads to a misinterpretation of the effect size: the shortcut estimator remarkably underestimates the effect size. This was to be expected from their general behavior in the case of radical discrepancy between the group sizes. That is, the “large” effect size (*d* = 0.78) turns out to be “very large” (*d* = 1.27) when the discrepancy between the group sizes is taken into account. In the cases where the discrepancy is moderate or small (*p*_*d*_ < 0.30 – 0.40), the shortcut estimator may give a very close approximation of the true value.

## Discussion, Suggestions for Practical Users, and Restrictions

### Main Results in a Nutshell

The starting point for this article was the observation that, Cohen’s *d* is one of the most commonly used effect size estimators when comparing two groups and when using point-biserial correlation. Using the relationship between Cohen’s *d* and Cohen’s *f*, several general forms of *d* have been derived. These general forms of *d* can be used to estimate the magnitude of differences between two or more means.

By using the general formulas derived in this article, the evaluation of the magnitude of effect sizes in the multiple means settings is more accurate when it comes to the traditional thresholds of the qualitative epithets “small,” “medium,” “high,” “very high,” and “huge” for the effect size, because the traditional thresholds for *f* are based on simplified formulas that assume equal numbers of cases in the groups being compared. In many settings related to analysis of variance, this has led to under-interpretation of the effect sizes when it comes to verbal epithets, because in practical settings where effect size are used, equal group sizes are a rare special case.

Correct and comparable forms for transforming the estimates of *f* to the scale of *d* for binary and dichotomous settings are available in Cohen’s (1988) classic book, but they do not seem to be in general use, judging from the fact that the simplified forms circulate in tutorial materials (e.g., Statistics How To, 2024; UCLA, 2021; Wikipedia, 2024; see however, https://www.psychometrica.de/effect_size.html) as well as in serious research papers (e.g., Correll et al., 2020; Gignac & Szoborai, 2016; Kim, 2016). This article provided a mechanism by which comparable effect sizes and associated thresholds are also available in the polytomous setting. The article also provided comparable thresholds for Cohen’s *f* and point-biserial correlation for the binary setting with selected proportions of cases in the subpopulations. The reader was also reminded of the comparable forms of calculation of *d* and *f*; these have puzzled scholars (see, e.g., Hartung et al., 2008; Hedges, 1981; Kraemer, 1983; McGrath & Meyer, 2006) to the extent that it has not been suggested that the verbal description be used as all (see, e.g., Correll et al., 2020).

### Suggestions for the Practical Users

To summarize the suggested procedure for using the generalized *d* to compare multiple groups (or to use precise effect size thresholds for Cohen’s *f* in the case of multiple means), the following steps should be taken:

1) Compute the estimate of eta squared (“*X dependent*”) between the grouping variable *g* and the dependent variable *X*.
2) Compute *f* either using the estimator in equation (9) which produces only the positive values, or, if you have an ordinal grouping variable, using equation (10) which also produces negative estimates. For this, you also need the correlation between the ordinal grouping variable *g* and the metric variable *X* (*R*_*gX*_). Note that the negative estimates only make sense with dichotomous, ordinal, and interval data sets.
3) Calculate the proportions of cases in the groups in your analysis (*p*_*i*_). These are needed to calculate an accurate estimate of the effect size.
4) To transform the estimates of *f* to the scale of *d*, use the formula $d_{11}=f/\sqrt{\frac{R}{4\left(R-1\right)}\overset{R}{\underset{i=1}{\sum }}p_{i}\left(1-p_{i}\right)}$ , where *R* is the number of subpopulations and *p*_*i*_ refers to the proportions of cases in the subpopulations.
5) Alternatively, if the group sizes are very close to each other or you have a dichotomous setting, you can use the traditional simple estimator *d* = 2*f* in transforming the *f* values to the scale of *d*. This formula always gives underestimations, but the underestimation may be nominal when the proportions of the cases are close to each other. If the discrepancy between the number of cases in the groups is medium to large (*p*_*d*_ > 0.40), this form leads to a radical underestimation.
6) When calculated the generalized *d*, use the standard benchmarks developed for Cohen’s *d*: approximately 0.1 for “very small,” 0.2 for “small,” 0.5 for “medium,” 0.8 for “large,” 1.2 for “very large,” and 2 for “huge” effect size.

### Known Restrictions and Some Possibilities for Further Research

Although the new formulas for *d* that apply when comparing multiple means are general in the sense that they are not restricted to a particular scale or number of categories, the estimates may still be debatable. The fact that the estimates give equal estimates does not mean that they are “correct.” However, we can note that (1) the basis of the formulas is justified, (2) the reduced forms fit the theory in the cases of equal group sizes as well as (3) in the special case of two means, (4) independent benchmarking shortcut estimators give broadly similar results, and (5) the result generally makes sense. Finally, and less seriously, (6) the formulas seem to be “something which has yet to fail in any obvious way” (Kaiser, 1970, p. 405, describing the feelings of the team after they introduced the famous Kaiser test for factor analysis in 1970). Systematic studies with the estimators would be beneficial.

The asymptotic or exact standard errors are not given for the new estimators. However, Cohen’s *d* based on *t*-test statistics, is known to follow a non-central *t* distribution (see, e.g., IBM, 2022). A reasonable assumption is that the generalized *d* based on the *f* statistic based on the *f* test statistic follows a non-central *f* distribution in some form. All terms in the formulas except *f* are constants or parallel. Therefore, the asymptotic standard errors could be computed based on this information.

The usefulness and error mechanisms associated with the shortcut estimators should be systematically studied with different data sets. Further studies in this regard is needed. A relevant research question is under what circumstances would the simple shortcut estimators be least susceptible to the apparent bias resulting from the discrepancy in the number of cases in the subpopulations?

The potential challenge of radical deflation in the values of eta squared and *R*_*gX*_ (see Metsämuuronen, 2022a, 2023a), which may cause radical deflation in the estimates of *d* and *f* (see Metsämuuronen, 2023b, 2023c) was not discussed further in this article. That is, the magnitude of the traditional estimates of *d* and *f* may be *much* too low because the correlation coefficient cannot reach the full range of values ranging from −1 to +1. In cases of extreme discrepancy between the number of cases in the subpopulations, the magnitude of the estimates of the coefficients eta and *R*_*gX*_ approximate zero even if the true correlation would be “perfect” $R_{gX}=\eta \left(g|X\right)=1$ (see simulations in Metsämuuronen, 2022b). As a consequence, PMC and eta may give much too low estimates of the association in the cases where the number of categories in two variables radically different as is always the case in the settings where *t*-test, point-biserial correlation, and eta squared are used (see Metsämuuronen, 2022a, 2022b, 2023a). Metsämuuronen (2023b, 2023c) discusses relevant deflation corrections for *d* and *f*. Systematic studies in this area would be beneficial.

The fact that the traditional thresholds for Cohen’s *f* are based on a special case of equal group sizes casts a shadow on the thresholds for other traditional effect size estimators based on multiple groups. Perhaps they are also based on simplistic assumptions? Of these, Cohen’s *w* in relation to the chi-squared statistic would be worth examining from this perspective. Also, Cohen himself noted that the transformation of the *r* effect size to the scale of *d* (or vice versa) assumes that the number of cases in the subpopulations is equal (Cohen, 1988, p. 82). Thus, the effect size thresholds related to the point-polyserial correlation (*R*_*PP*_), that is, 0.1, 0.3, and 0.5 for “small,” “medium,” and “large,” respectively, are based on the simplified assumption of equal subpopulation sizes and, most likely, on an incorrect transformation formula of point-biserial correlation to biserial correlation (see Cohen, 1988, p. 82). The effect of different subpopulation sizes needs to be systematically studied also in polytomous settings.

Simple rules of thumb are sometimes needed in practical research settings. However, in the case of effect sizes, and especially in the case of Cohen’s *f*, *r*, and eta squared, the overly simple traditional rules can lead to evaluate effect sizes to be far too small. A size of *f* = 0.25, traditionally called “medium,” may turn out to be “very large,” depending on the proportions of cases in the subpopulations, as noted in the dichotomous settings. This phenomenon generalizes to the shortcut estimators derived in the article: if Cohen’s *f* is inadequately transformed to the scale of *d*, the effect size may be radically underestimated by the common *d*.

Finally, we may recall the quote from Guttman (1945, p. 260) regarding estimates of test reliability: “Reliability has often been underestimated by the conventional formula […]. Many tests are more reliable than they have been considered.” By starting to use proper formulas for transforming estimates of *f* and *r* to the scale of *d*, or by re-evaluating old results, we may come to the same conclusion with effect sizes: “Effect sizes have often been underestimated by the conventional formulas. In many cases, the difference between the means should be considered larger and the relationship between two variables should be considered higher than it has been considered.”

## Supplemental Material


Supplemental Material - Generalized Cohen’s d for Multiple Means and Polytomous Settings
Supplemental Material for Generalized Cohen’s d for Multiple Means and Polytomous Settings by Jari Metsämuuronen in Applied Psychological Measurement.

## Data Availability

The dataset used in the empirical section of the article is available in CSV format at https://doi.org/10.13140/RG.2.2.20359.57762/1 and in IBM SPSS format at https://doi.org/10.13140/RG.2.2.33781.35042/1 (Metsämuuronen, 2024a, 2024b).

## Note

1. In this form, we compute all possible combinations, and then *p*_*i*_*p*_*j*_ is different from *p*_*j*_*p*_*i*_. Alternatively, we could compute all the elements of *i* < *j*. Then, the number of elements would be 0.5 *R* (*R* − 1).
