# Supplementary material for: Generalized Cohen’s d for Multiple Means and Polytomous Settings
Source: Appl Psychol Meas. 2026 Jan 20:01466216261416025. Online ahead of print. doi: 10.1177/01466216261416025 (PMC12819128; doi:10.1177/01466216261416025)
Supplement: Supplemental Material - Generalized Cohen’s d for Multiple Means and Polytomous Settings [file sj-pdf-1-apm-10.1177_01466216261416025.pdf]

Appendix 1. Dataset used in simulation of short-cut options

The original dataset with 30 multiple-choice questions (MCQ) with binary outcome and 4,023 test takers was based on settings related to test of achievement in mathematics (FINEEC, 2018). In the original dataset, the proportions of cases in the subpopulation 1 in binary sub-populations ranged $0.24 < p < 0.95$ with the average of $\overline{p} = 0.63$, item-score correlations between g and X varied $0.332 < R_{gX} < 0.627$ with the average of $\overline{R_{gX}} = 0.481$. The reliability in the original dataset was reasonably high ($\alpha = 0.885$) which implies that the item–score correlations (R_{gX} and *eta*) were relatively high. Hence, also in the outcome, most of the estimates by *d* and *f* are relatively high. For the dataset used in the modelling, items were both used as they were, and they combined and remerged so that it also includes polytomous variables. Mechanism for this is discussed later, and it has an impact on the interpretation or generalization of the results.

The dataset is available in CSV format at http://dx.doi.org/10.13140/RG.2.2.20359.57762/1 and in IBM SPSS format at http://dx.doi.org/10.13140/RG.2.2.33781.35042/1.

The simulation dataset was formed as follows. First, ten finite random samples of $n = 25$, 50, 100, and 200 test-takers were drawn from the original dataset; these represent real-life settings of small-scale sampling or testing in classrooms to larger student groups. In each of the $10 \times 4 = 40$ datasets, second, 36 shorter tests were produced by varying the number of items (k), difficulty levels of the items (p_g), number of categories in the item (R), and in the score (C). As a result, the dataset consisted of 14,880 partly related test items from 1,440 tests. Because the interest in in the polytomous settings, these interest specifically. The dataset consists of 6,932 polytomous items with a varying number of test-takers ($n = 25$, 50, 100, and 200), the difficulty levels of the items (p_g = 0.32–0.91; $\overline{p_g} = 0.66$; SD = 0.077) and of the score variable (p_X = 0.50–0.76; $\overline{p_X} = 0.66$; SD = 0.0497), discrepancy between the group sizes for the polytomous items (p_d = 0.11–0.84; $\overline{p_d}$ = 0.41; SD = 0.131), number of categories in the items (R = 3–16; $\overline{R} = 4.78$; SD = 2.501) and in the score variable (C = 10–27; $\overline{C} = 18.28$; SD = 3.985), and the item–score correlations (R_{gX} = 0.03–0.97; $\overline{R_{gX}} = 0.71$; SD = 0.139, $\eta_{g|X}$ = 0.17–0.99; $\overline{\eta_{g|X}} = 0.73$; SD = 0.137, and $\eta^2_{g|X}$ = 0.03–0.99; $\overline{\eta^2_{g|X}} = 0.55$; SD = 0.194). Even if negative estimates by *f* and *d* were also available, they were observed only in the binary settings.

Three specific characteristics of the dataset is worth highlighting. First, in the measurement modelling settings—unlike the general settings—the higher gets the number of categories in items and the less these items are, the higher is the correlation between an item and the score approximating $R_{gX} = 1$ in the case that the number of categories in the item approximates infinity. The reason for this phenomenon is that, in the measurement modelling settings, the score is a compilation of individual items, and they are technically related. Therefore, in the dataset, the magnitude of the effect sizes gets the higher the more categories there are in the item.

Second, the mechanism of producing the polytomous items makes their number notably smaller than those of dichotomous items. Namely, for each score, several “items” with different numbers of categories were formed. For instance, with 30 binary items (categories 0–1; $R = 2$), 15 items with three categories (0–1–2; $R = 3$) were formed by summing up every 15th item. Parallel, 10 items with four categories (0–1–2–3; $R = 4$) were formed by summing up every tenth item, six items with

five categories (0–1–2–3–5; R = 6) and five items with six categories (0–1–2–3–5–6; R = 7) were formed by summing up every sixth and fifth item, respectively. By summing up every third item, 3 items with 11 categories (0–1–2–3–5–6–7–8–9–10; R = 11) were formed and, finally, by summing up every second item we get 2 items with 16 categories (0–1–2–3–5–6–7–8–9–10–11–12–13–14–15; R = 16). Hence, as an outcome with this set of items we get 30+15+10+6+5+3+2 = 71 items of which the binary ones dominate the dataset in frequency, and, within the polytomous items, most of the estimates (60%) come from the settings with 3 or 4 groups.

Third, in the polytomous settings, a crucial concept is the discrepancy between the number of cases in the sub-populations. This is indicated by an index or discrepancy (p_d) computed simply as the absolute difference between the proportions of the groups with the highest and lowest number of cases in the sub-population. The value $p_d = 0$ means that the group sizes are equal and the wider the discrepancy, the higher the value approximating $p_d = 1$. In the dataset, the most extreme discrepancies were observed with groups with a small number of sub-populations. Contrarily, the settings with many sub-populations ($R > 7$) tended to have fewer cases with extreme discrepancy between the number of cases. This means that, in the dataset, there is a tendency that, by the increasing number of groups, the setting gets closer to the situation where $p_d = 0$ and, hence, where the simple estimator $d = 2f$ gives accurate estimates.

Table 1 collects the number of items and selected characteristics of the dataset and relevant characteristics related to the general and short-cut estimators for the generalized d are collected in Tables 2 and 3. Notably, regardless of the specificities in the dataset, these do not have effect on estimating Cohen's f because the underlying coefficient eta do not use this information in computing. The estimates just are very high because of the high correlations.

Table 1. Selected statistics from the dataset used in the simulation

	R	p(g) item difficulty	R_{gX} item-score correlation	Eta2 eta squared	Cohen d	Cohen f	N (observed R)	N (maximal R)
	2	0,6573	0,4730	0,2424	1,2455	0,5624	7948	7,920
	3	0,6600	0,6123	0,4017		0,8364	3056	3,000
	4	0,6635	0,7007	0,5210		1,0735	1390	1,360
	5	0,6603	0,7659	0,6184		1,3179	729	720
	6	0,6439	0,8092	0,6880		1,5468	474	400
	7	0,6667	0,8477	0,7492		1,7954	366	360
	8	0,6570	0,8776	0,7992		2,0964	255	280
	9	0,6752	0,9005	0,8395		2,4287	139	120
	10	0,6497	0,9157	0,8641		2,7223	160	120
	11	0,6430	0,9287	0,8817		2,9229	136	200
	12	0,6695	0,9407	0,9034		3,2508	92	80
	13	0,6751	0,9441	0,9090		3,3501	68	80
	14	0,6736	0,9439	0,9034		3,0959	42	80
	15	0,6587	0,9463	0,9062		3,1569	21	80
	16	0,6446	0,9445	0,8992		3,0065	4	80
Mean	3,2900	0,6585	0,5842	0,3840	1,2455	0,8944		
Std. Dev.	2,2000	0,1073	0,1820	0,2192	0,4457	0,6220		
Total							14,880	14,880

Table 2. Different estimators of d in the polytomous settings (R > 2)

		Mean			Std. Deviation		
R	N	d6 = d11 exact	d7= d12 exact	d15 = 2f short-cut	d6 = d11 exact	d7= d12 exact	d15 = 2f short-cut
3	3056	1,7801	1,7801	1,6727	0,4742	0,4742	0,4530
4	1390	2,2547	2,2547	2,1469	0,5290	0,5290	0,5079
5	729	2,7399	2,7399	2,6359	0,6108	0,6108	0,5945
6	474	3,1815	3,1815	3,0937	0,7060	0,7060	0,6920
7	366	3,6889	3,6889	3,5908	0,7946	0,7946	0,7798
8	255	4,2918	4,2918	4,1928	0,9848	0,9848	0,9578
9	139	4,9648	4,9648	4,8574	1,2209	1,2209	1,2050
10	160	5,5389	5,5389	5,4446	1,5218	1,5218	1,4963
11	136	5,9331	5,9331	5,8458	1,5691	1,5691	1,5428
12	92	6,6029	6,6029	6,5015	1,6571	1,6571	1,6271
13	68	6,8069	6,8069	6,7002	1,8867	1,8867	1,8515
14	42	6,2948	6,2717	6,1918	0,6522	0,6336	0,6278
15	21	6,3917	6,3917	6,3138	0,7720	0,7720	0,7598
16	4	6,0819	6,0819	6,0131	0,4768	0,4768	0,4771

1) allows and includes negative values; in the dataset, however, these were observed only in the binary settings

Table 3. Different estimators of d by the discrepancy index p_d (R > 2)

		mean		standard deviation	
p_d[1]	N	d6 = d11 = d7[2] =d12[2] exact	d15 = 2f short-cut	d6 = d11 = d7 = d12 exact	d15 short-cut
>0.0 - <0.2	1185	4,1246	4,0711	2,0474	2,0170
0.2 - <0.3	2272	2,8206	2,7448	1,2930	1,2610
0.3 - <0.4	1887	2,1324	2,0303	0,7335	0,7001
0.4 - <0.5	1096	2,0109	1,8539	0,6056	0,5618
0.5 - <0.60	345	1,7925	1,5790	0,5406	0,4765
0.6 - <0.90	147	1,7491	1,4362	0,5089	0,4104

1) discrepancy index = p_{max} - p_{min}; difference between the greatest and lowest proportion of number of cases in the sub-populations
2) allows and includes negative values; however, in the simulation dataset, these were observed only in the binary settings
